# Characterization of the Functional Cross-Talk between Surface GABA_A_ and Dopamine D5 Receptors

**DOI:** 10.3390/ijms22094867

**Published:** 2021-05-04

**Authors:** François Maingret, Laurent Groc

**Affiliations:** 1 Interdisciplinary Institute for Neuroscience, Université de Bordeaux, UMR 5297, 33076 Bordeaux, France; laurent.groc@u-bordeaux.fr; 2 CNRS, Interdisciplinary Institute for Neuroscience, UMR 5297, 33076 Bordeaux, France

**Keywords:** GABAA receptor, dopamine, hippocampus, synapse, single nanoparticle tracking, lateral diffusion, long-term potentiation, synaptic transmission, synaptic plasticity

## Abstract

The γ-aminobutyric acid type A receptor (GABA_A_R) plays a major role in fast inhibitory synaptic transmission and is highly regulated by the neuromodulator dopamine. In this aspect, most of the attention has been focused on the classical intracellular signaling cascades following dopamine G-protein-coupled receptor activation. Interestingly, the GABA_A_R and dopamine D5 receptor (D5R) have been shown to physically interact in the hippocampus, but whether a functional cross-talk occurs is still debated. In the present study, we use a combination of imaging and single nanoparticle tracking in live hippocampal neurons to provide evidence that GABA_A_Rs and D5Rs form dynamic surface clusters. Disrupting the GABA_A_R–D5R interaction with a competing peptide leads to an increase in the diffusion coefficient and the explored area of both receptors, and a drop in immobile synaptic GABA_A_Rs. By means of patch-clamp recordings, we show that this fast lateral redistribution of surface GABA_A_Rs correlates with a robust depression in the evoked GABAergic currents. Strikingly, it also shifts in time the expression of long-term potentiation at glutamatergic synapses. Together, our data both set the plasma membrane as the primary stage of a functional interplay between GABA_A_R and D5R, and uncover a non-canonical role in regulating synaptic transmission.

## 1. Introduction

γ-aminobutyric acid (GABA) is the main inhibitory neurotransmitter in the central nervous system. Fast inhibitory GABAergic synaptic transmission acts through the activation of the GABA_A_ receptors (GABA_A_Rs), which are heteropentameric ligand-gated chloride-permeable channels [1,2]. GABA_A_Rs play a crucial role in the balance between the inhibitory and excitatory transmission that controls neuronal network activity, and consequently any impairment of GABAergic synaptic transmission leads to neurological disorders [3,4]. In mammals, up to 19 subunits—6α, 3β, 3γ, 3⍴, δ, ε, π, θ—associate to form functional GABA_A_Rs [5], with distinct gating/pharmacological properties that are differentially expressed in several brain areas [6,7]. Noteworthy, despite this considerable functional diversity, most GABA_A_Rs expressed in the brain are composed of 2α, 2β and 1γ subunits [8]. To play their role, GABA_A_Rs have to be expressed and clustered at the inhibitory postsynaptic membrane but, contrary to excitatory synapses, inhibitory synapses lack a prominent post synaptic density (PSD). This scaffolding apparatus anchors glutamate receptors at the excitatory postsynaptic membrane and links them to intracellular regulatory proteins to mediate excitatory neurotransmission [9]. At the inhibitory synapses, the tubulin-binding protein gephyrin has been identified to be a major component of the scaffolding matrix and essential to cluster postsynaptic GABA_A_Rs [10,11]. More importantly, the clustering and long-lasting maintenance of GABA_A_Rs at the inhibitory postsynaptic membrane does not only require gephyrin but also the γ2-GABA_A_ subunit, even if no direct binding has been reported [12,13,14]. Postsynaptic GABA_A_Rs undergo a constant turnover to maintain the efficacy of the fast inhibitory GABAergic transmission. This process is regulated by scaffolding proteins, but also by the exocytosis of de novo synthetized GABA_A_Rs or the endocytosis of recycled ones [15]. In addition, several lines of evidence point that a much faster turnover can occur through the lateral diffusion of membrane GABA_A_Rs [16]. The distribution and stability of postsynaptic GABA_A_Rs are regulated by intracellular signaling pathways following G-protein-coupled receptor (GPCRs) activation. This functional interplay between GPCRs and GABA_A_Rs regulates GABA_A_Rs channel activity and involves the direct phosphorylation/dephosphorylation of γ2-GABA_A_ and β1–3 GABA_A_ subunits [15,17]. Interestingly, in most cases, the GPCR–GABA_A_R interplay leads to a decrease in the receptor activity upon the activation of the associated receptors. Such cross-talks have been described for GABA_B_ receptors [18] and adenosine A1 receptors [19] but the most documented are the ones between the GABA_A_Rs and dopamine receptors [20,21,22,23,24]. Dopamine is probably the most versatile neuromodulator in the brain and has a prominent role in regulating synaptic transmission [25]. Five subtypes of dopamine receptors have been identified and divided into D1-like (D1 and D5) and D2-like (D2, D3 and D4) receptor families based on their coupling to either G_α,olf_ or G_αi/o_ which respectively stimulates or inhibits the production of cAMP [26,27]. Interestingly, although a functional cross-talk has been reported for the GABA_A_R and almost all dopamine receptors, only the dopamine D5 receptor (D5R) physically interacts with the GABA_A_R [28]. This physical interaction has been originally demonstrated in the hippocampal tissues and involves the second intracellular loop of the γ2-GABA_A_ subunit and the C-terminal domain of D5R. As previously mentioned, D1R and D5R form the dopamine D1-like family and share strong homologies in their transmembrane domains [27]. D5R only differs from D1R in its C-terminal domain and by a 10-fold higher affinity for dopamine [27,29]. Regarding its distribution profile, D5R is widely expressed in the whole hippocampus while D1R is more restricted to the dentate gyrus [30,31,32]. When expressed in pyramidal neurons, D1R is enriched near the spines and D5R in the dendritic shafts [32,33,34]. Functionally, the physical interaction between the GABA_A_R and the D5R exerts a reciprocal inhibitory effect, independent of intracellular signaling, as dopamine decreases GABA_A_R-mediated currents while GABA decreases cAMP production [28]. However, this functional cross-talk was only demonstrated in heterologous expression systems and whether such a functional interplay between the GABA_A_R and D5R occurs in hippocampal neurons remains an open question. In the present report, we demonstrate, using a combination of imaging, single nanoparticle tracking and electrophysiology, that the GABA_A_R–D5R interaction affects both the receptors localization and surface dynamics but also alters GABAergic synaptic transmission and plasticity in live hippocampal neurons.

## 2. Results

### 2.1. Organization of γ2-GABA_A_ Receptors Clusters in Hippocampal Neurons

**Figure 1:**
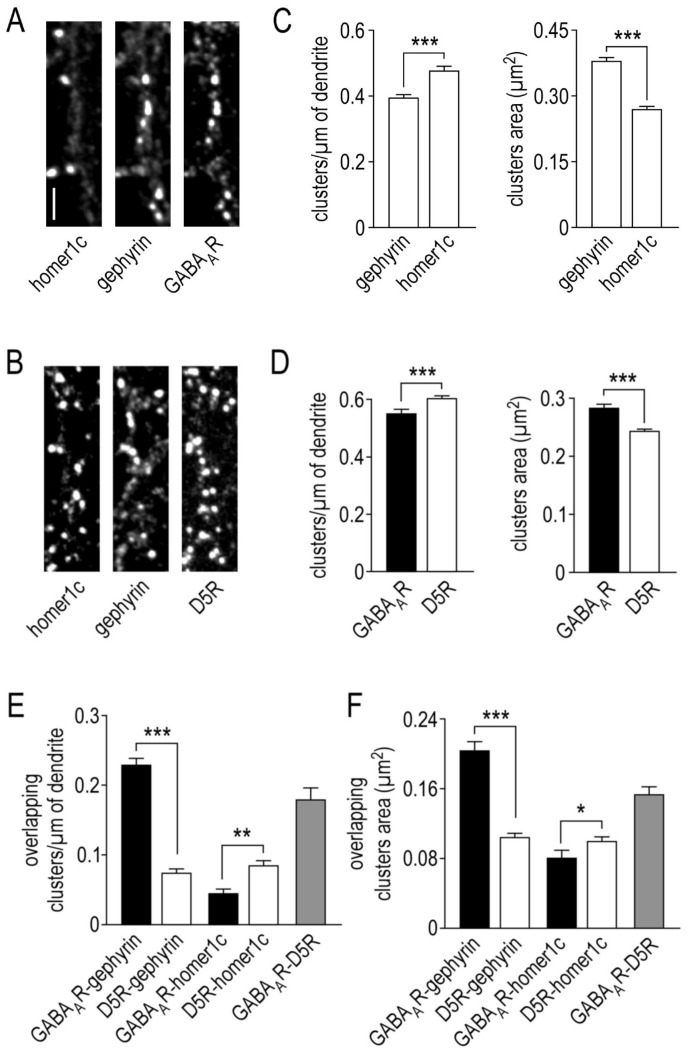
Immunolocalization of GABA_A_ and dopamine D5 receptors in hippocampal neurons. Representative images of GABA_A_ receptors (**A**) and dopamine D5 receptors (**B**) surface staining and their dendritic localization relative to excitatory and inhibitory synaptic areas (identified respectively by homer1c and gephyrin). Scale bar, 4 µm. (**C**) Bar graphs illustrating homer1c and gephyrin clusters linear density and mean area (left and right panels, respectively). (**D**) Bar graphs illustrating GABA_A_ receptors and dopamine D5 receptors clusters linear density and mean area (left and right panels, respectively). Bar graphs illustrating the linear density (**E**) and the mean area (**F**) of overlapping clusters of GABA_A_ receptors with gephyrin and homer1c or dopamine D5 receptors with gephyrin, homer1c and GABA_A_ receptors. Data are represented as mean ± SEM. * *p* < 0.05, *** *p* < 0.001. Nonparametric Mann–Whitney test.

The γ2-GABA_A_ subunit and the D5R were respectively tagged with a superecliptic pHluorin (SEP) or a yellow fluorescent protein (YFP), expressed in cultured hippocampal neurons and characterized after 14–16 days in vitro (DIV) by immunocytochemistry, using antibodies directed against the GFP. In parallel, the endogenous expression of homer1c (postsynaptic compartment of glutamatergic synapses) and gephyrin (postsynaptic compartment of GABAergic synapse) proteins were characterized to assess the synaptic localization of the receptors (Figure 1A,C). Figure 1A–C show that gephyrin and homer1c have a different expression profile. At 14–16 DIV, the linear density of homer1c (0.478 ± 0.012 clusters/µm of dendrite, *n* = 89 neurons) is significantly higher compared to gephyrin (0.396 ± 0.009, *n* = 165; *p* < 0.001). This is associated with a significantly smaller cluster size (0.270 ± 0.007 and 0.380 ± 0.007 µm^2^, *p* < 0.001) without any difference in the cluster intensity (data not shown). Figure 1D shows that the clusters of γ2-GABA_A_Rs have an area of 0.283 ± 0.006 µm^2^ and a linear density of 0.554 ± 0.014 (*n* = 166). By comparison, the cluster number of D5Rs is significantly greater (0.606 ± 0.009 clusters/µm of dendrite) but smaller (0.243 ± 0.003 µm^2^, *n* = 220, *p* < 0.001) without any difference in cluster intensity (data not shown). The synaptic clusters were then evaluated as γ2-GABA_A_R and D5R clusters that co-localized with the synaptic markers (Figure 1E,F). As expected, γ2-GABA_A_Rs are highly expressed at inhibitory synapses (0.232 ± 0.008 overlapping clusters/µm of dendrite, *n* = 86) and their surface area is large (0.205 ± 0.009 µm^2^). As a negative control, we also assessed the expression of γ2-GABA_A_Rs at excitatory synapses. Not surprisingly, the linear density is low (0.048 ± 0.003 overlapping clusters/µm of dendrite, *n* = 14) and when detected, the surface area of the overlapping clusters is very small (0.081 ± 0.008 µm^2^).

### 2.2. Organization of Dopamine D5 Receptors Clusters in Hippocampal Neurons

We then took advantage of this comparison to investigate the synaptic distribution of dopamine D5 receptors (Figure 1E,F). First, D5Rs are weakly expressed at excitatory synapses (0.086 ± 0.006 overlapping clusters/µm of dendrite, *n* = 75) but significantly higher compared to γ2-GABA_A_Rs (*p* = 0.004). More interestingly, D5Rs are not enriched at inhibitory synapses either (0.075 ± 0.005 overlapping clusters/µm of dendrite, *n* = 79), and are significantly lower compared to the high density of γ2-GABA_A_Rs (*p* < 0.001). Finally, the overlapping clusters area of D5Rs is relatively small, both with homer1c (0.101 ± 0.004 µm^2^) and gephyrin (0.105 ± 0.004 µm^2^) proteins.

### 2.3. Physical Interaction between γ2-GABA_A_ and Dopamine D5 Receptors

**Figure 2:**
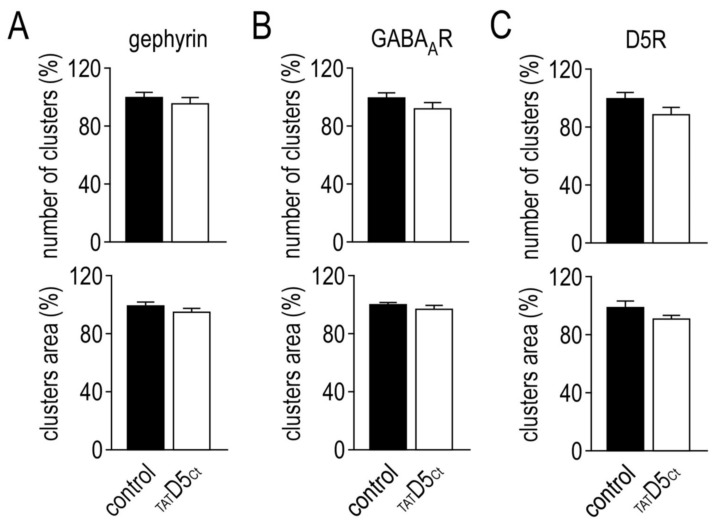

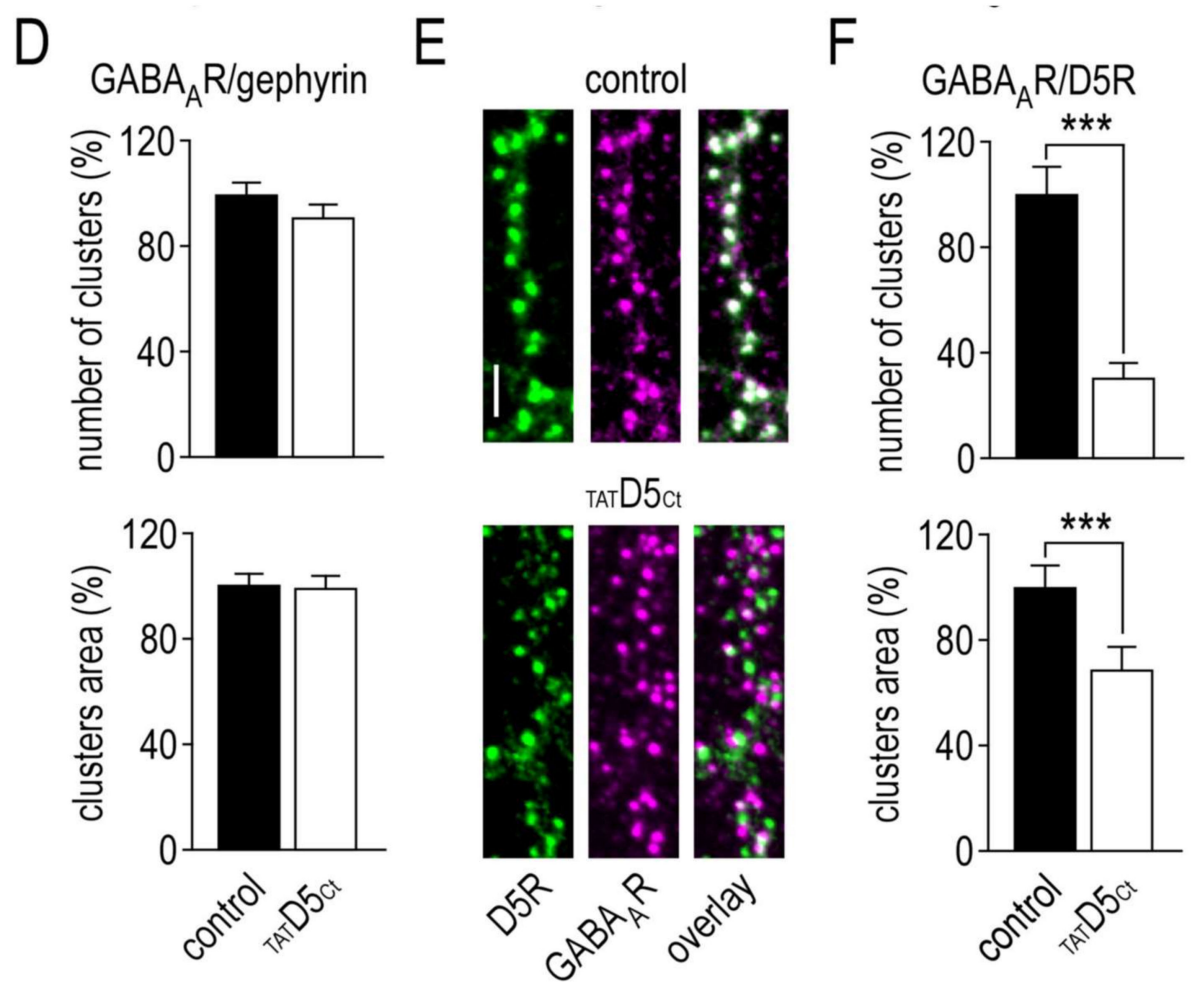
The disrupting peptide impairs the co-localization of GABA_A_ and dopamine D5 receptors in hippocampal neurons. Bar graphs illustrating the impact of the disrupting peptide on the number and the mean area of the clusters of gephyrin (**A**), GABA_A_ receptors (**B**) and dopamine D5 receptors (**C**). (**D**) Bar graphs illustrating the impact of the disrupting peptide on the synaptic GABA_A_ receptors. (**E**) Representative images of dopamine D5 receptors (green) and GABA_A_ receptors (purple) in control condition (upper panel) or in the presence of the disrupting peptide (lower panel). The overlapping clusters appear in white. Scale bar, 4 µm. (**F**) Bar graphs illustrating the impact of the disrupting peptide on the overlapping clusters of GABA_A_ and dopamine D5 receptors. Data are represented as mean ± SEM normalized to the control condition. *** *p* < 0.001. Nonparametric Mann–Whitney test.

Next, we investigated the co-localization of D5Rs with γ2-GABA_A_Rs. To this aim, we performed immunocytochemical experiments in which the extracellular tag SEP was swapped with a myc tag. We provide evidence that γ2-GABA_A_Rs and D5Rs co-localized with a linear density of 0.181 ± 0.016 overlapping clusters/µm of dendrite (Figure 1E) and an overlapping clusters area of 0.155 ± 0.007 µm^2^, *n* = 66 (Figure 1F) (also illustrated in Figure 2E). Since D5Rs are weakly concentrated at both the excitatory or inhibitory synapses, and since they interact with γ2-GABA_A_Rs, it suggests that this interplay mostly occurs on the dendritic shaft at extrasynaptic sites.

To further investigate whether this interaction between γ2-GABA_A_Rs and D5Rs shapes the distribution of the receptors, we disrupted the γ2-GABA_A_R–D5R complexes using a cell-permeant (TAT-conjugated) peptide (_TAT_D5_Ct_) mimicking the C-terminal sequence of the D5R required for the interaction with the γ2-GABA_A_ subunit [28]. First, we investigated the impact of the disrupting peptide (10 µM, 45 min) on the inhibitory synaptic marker gephyrin. Figure 2A shows that it had no impact neither on the number of clusters (control: 1 ± 0.03, *n* = 47; _TAT_D5_Ct_: 0.96 ± 0.03, *n* = 57) nor on the cluster area (control: 1 ± 0.04; _TAT_D5_Ct_: 0.89 ± 0.04) of gephyrin. Second, we investigated its impact on the γ2-GABA_A_Rs and D5Rs. The disrupting peptide had no impact on the characteristics of neither the individual clusters of γ2-GABA_A_Rs (linear density, control: 1 ± 0.22, *n* = 64; _TAT_D5_Ct_: 0.93 ± 0.03, *n* = 82; cluster area, control: 1 ± 0.02; _TAT_D5_Ct_: 0.97 ± 0.02) nor D5Rs (linear density, control: 1 ± 0.04, *n* = 17; _TAT_D5_Ct_: 0.89 ± 0.06, *n* = 25; cluster area, control: 1 ± 0.04; _TAT_D5_Ct_: 0.91 ± 0.02) (Figure 2B,C). Synaptic γ2-GABA_A_Rs were not affected by the treatment either (Figure 2D, linear density, control: 1 ± 0.05, *n* = 47; _TAT_D5_Ct_: 0.91 ± 0.05, *n* = 57; cluster area, control: 1 ± 0.04; _TAT_D5_Ct_: 0.99 ± 0.04). Then, we quantified its impact on the co-localized γ2-GABA_A_Rs and D5Rs (Figure 2E, upper panel). Strikingly, both the number of clusters (control: 1 ± 0.1, *n* = 17; _TAT_D5_Ct_: 0.31 ± 0.06, *n* = 25; *p* < 0.001) and the cluster area (control: 1 ± 0.08; _TAT_D5_Ct_: 0.68 ± 0.08, *p* < 0.001) were strongly affected by the disrupting peptide (Figure 2E,F). The latter has no effect on the individual γ2-GABA_A_R and D5R clusters but only on the interacting ones, further demonstrating its efficiency.

### 2.4. Surface Dynamics Properties of γ2-GABA_A_ Receptors

**Figure 3:**
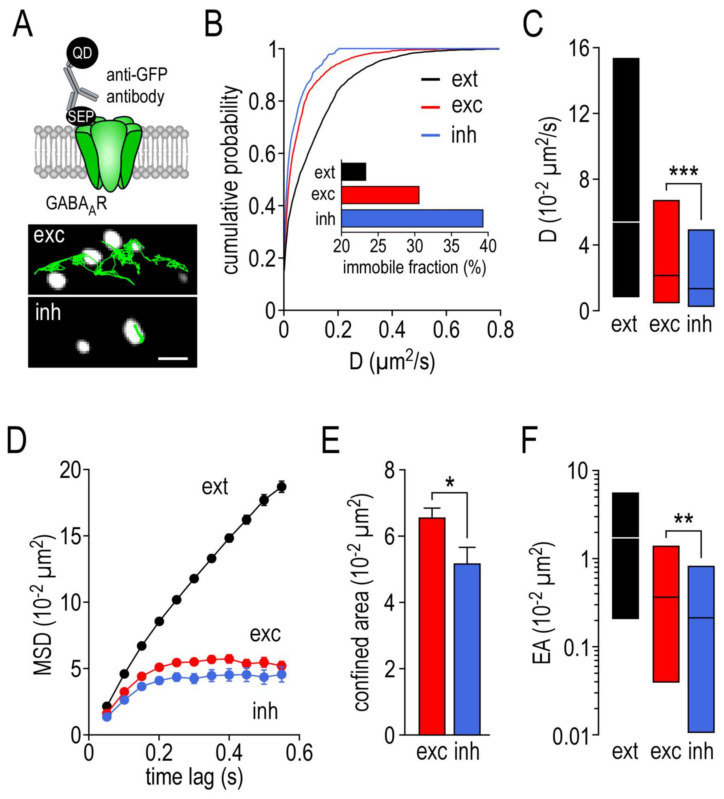
The surface dynamics properties of GABA_A_ receptors at inhibitory synapses differ from the excitatory synapses. (**A**) Cartoon showing the experimental design (upper panel) and examples of trajectories of a single GABA_A_ receptor–SEP–anti-GFP antibody–QD complex (500 frames, 50 ms acquisition) on the dendritic shaft with either identified excitatory (exc) or inhibitory (inh) synaptic areas (lower panel). Scale bar, 350 nm. (**B**) Cumulative probability diffusion coefficient of GABA_A_ receptors at inh or exc synapses compared to extrasynaptic area (ext). The inset shows the immobile fraction (defined as D < 0.005 µm^2^/s) in the different areas. (**C**) Bar graphs illustrating the instantaneous diffusion coefficient (D, represented as median ± interquartile range 25–75%) in ext, exc and inh areas. (**D**) Comparison of GABA_A_ receptors mean square displacements (MSD, represented as mean ± SEM) in ext, exc and inh areas. (**E**) bar graphs illustrating the confined area (for the time period of 250–550 ms and represented as mean ± SEM) in exc and inh areas. (**F**) Bar graphs illustrating the explored surface area (EA, represented as median ± interquartile range 25–75%) in ext, exc and inh areas. * *p* < 0.05, ** *p* < 0.01, *** *p* < 0.001. Nonparametric Mann–Whitney test.

We then used single nanoparticle tracking to investigate the live surface distribution and lateral diffusion of the receptors and evaluate the impact of the disrupting peptide on their surface dynamics. These experiments were performed at 14–16 DIV on cultured hippocampal neurons transfected with the γ2-GABA_A_ subunit fused with a SEP tag. The complex formed by the membrane receptor–anti-GFP antibody–quantum dots (Figure 3A, upper panel) was tracked at the surface of the live neurons using single nanoparticle detection. The excitatory postsynaptic locations were determined by the co-transfection of the neurons with homer1c–mDsRed. We also took advantage of the unique property of SEP, which only emits fluorescence at neutral pH. As only the fluorescence of the SEP γ2-GABA_A_Rs expressed at the plasma membrane is detected, it served as a reporter of the inhibitory postsynaptic locations. In such experiments, the surface dynamic properties of SEP-γ2-GABA_A_Rs are similar to those where the identification of the inhibitory postsynaptic locations was performed using the synaptic marker gephyrin–mRFP (Supplementary Figure S1). Thus, each trajectory of the surface of the SEP-γ2-GABA_A_Rs could be correlated, in the same experiment, to the excitatory (exc), inhibitory (inh) and extrasynaptic (ext) sites (as illustrated in Figure 3A, lower panel). The SEP-γ2-GABA_A_R diffusion was lower at the synaptic sites, as demonstrated by the left shift of the distribution curves (Figure 3B). Noteworthy, the left shift of the synaptic distribution is more pronounced at the inhibitory than at the excitatory synaptic sites. Consistently, the SEP-γ2-GABA_A_R diffusion coefficient median at the inhibitory synaptic sites (Figure 3C, 1.37 ± IQR 0.27 to 4.92 10^−2^ µm^2^/s, *n* = 422) was significantly reduced when compared to the one at the excitatory synaptic sites (2.17 ± IQR 0.48 to 6.72 10^−2^ µm^2^/s, *n* = 1652, *p* < 0.001). Both of these values were lower than the ones at the extrasynaptic sites (5.47 ± IQR 0.85 to 15.38 10^−2^ µm^2^/s, *n* = 3800). When we calculated the immobile fraction (D < 0.005 µm^2^/s), it appeared that the SEP γ2-GABA_A_Rs were much more immobile at the inhibitory synapses than at the excitatory synapses, and at the extrasynaptic sites (Figure 3B inset). To further explore the behavior of SEP-γ2-GABA_A_Rs, we examined the mean square displacement (MSD) over time, which reflects the confinement of the tracked receptors. Figure 3D shows that SEP-γ2-GABA_A_Rs exhibit a strong confinement level at both the excitatory and inhibitory synaptic sites compared to the extrasynaptic sites. However, the confined area is smaller at the inhibitory (Figure 3E, 5.24 ± 0.39 10^−2^ µm^2^, *n* = 316) than at the excitatory synapses (6.55 ± 0.26 10^−2^ µm^2^, *n* = 1216, *p* = 0.02). The confinement level is also correlated to the explored area. At the extrasynaptic sites, the explored area is large (1.63 ± IQR 0.21 to 5.60 10^−2^ µm^2^/s, *n* = 3387) and it is reduced at synaptic sites (Figure 3F). However, the reduction in the explored area was more pronounced at the inhibitory (0.20 ± IQR 0.01 to 0.82 10^−2^ µm^2^/s, *n* = 265) than at the excitatory synapses (0.20 ± IQR 0.04 to 1.32 10^−2^ µm^2^/s, *n* = 1030, *p* = 0.0049). Altogether, these data demonstrate that the SEP γ2-GABA_A_R surface distribution and lateral diffusion are regulated—a reduced diffusion coefficient and explored area, higher immobile fraction and confinement level—when they enter synaptic sites, particularly those of inhibitory synapses.

### 2.5. Impact of the Disrupting Peptide on the Surface Dynamics of γ2-GABA_A_ Receptors

**Figure 4:**
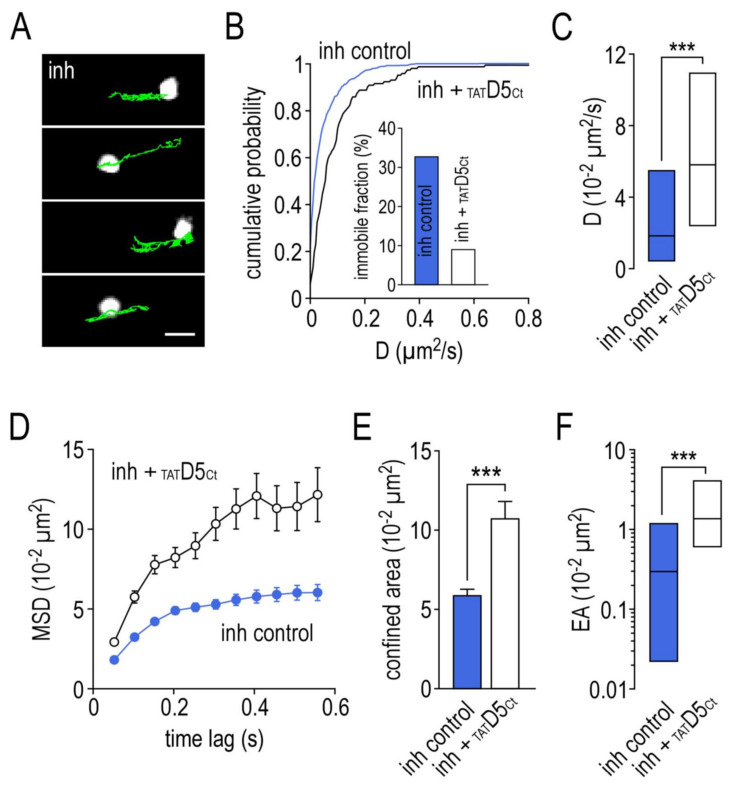
The disrupting peptide impairs the surface dynamic properties of GABA_A_ receptors at inhibitory synapses. (**A**) Examples of trajectories of a single GABA_A_ receptor–SEP–anti-GFP antibody–QD complex (500 frames, 50 ms acquisition) in the presence of the competing peptide (10 µM) with identified inhibitory (inh) synaptic areas. Scale bar, 350 nm. (**B**) Cumulative probability diffusion coefficient of GABA_A_ receptors at inhibitory synapses in control condition or in the presence of the disrupting peptide. The inset shows the immobile fraction (defined as D < 0.005 µm^2^/s) in the inhibitory area in control condition and in the presence of the disrupting peptide. (**C**) Bar graphs illustrating the instantaneous diffusion coefficient (D, represented as median ± interquartile range 25–75%) in inhibitory area in control condition or in the presence of the disrupting peptide. (**D**) Comparison of GABA_A_ receptors mean square displacements (MSD, represented as mean ± SEM) in inhibitory area in control condition or in the presence of the disrupting peptide. (**E**) Bar graphs illustrating the confined area (for the time period of 250–550 ms and represented as mean ± SEM) in inhibitory area in control condition or in the presence of the disrupting peptide. (**F**) Bar graphs illustrating the explored surface area (EA, represented as median ± interquartile range 25–75%) in inhibitory area in control condition or in the presence of the disrupting peptide. *** *p* < 0.001. Nonparametric Mann–Whitney test.

We then investigated the impact of the γ2-GABA_A_R–D5R complex on the receptors dynamics. Remarkably, incubating the hippocampal neurons with the disrupting peptide (10 µM, 10 min) prior to single nanoparticle tracking experiments drastically changed the surface dynamic properties of SEP-γ2-GABA_A_Rs at the inhibitory synapses (Figure 4A). The disrupting peptide shifted the distribution curve of the coefficient diffusion toward higher values (Figure 4B). As a consequence, the immobile fraction decreased from 33% in the control condition to 9% after the disrupting peptide incubation (Figure 4B, inset). The SEP -γ2-GABA_A_Rs were significantly more mobile at the inhibitory synaptic sites (Figure 4C, 5.78 ± IQR 2.37 to 10.88 10^−2^ µm^2^/s, *n* = 140) compared to the control condition (1.81 ± IQR 0.43 to 5.42 10^−2^ µm^2^/s, *n* = 644, *p* < 0.001). The disrupting peptide strongly decreased the confinement level at the inhibitory synapses (Figure 4D). The confined area was increased (Figure 4E, control: 5.93 ± 0.31 10^−2^ µm^2^/s, *n* = 508; _TAT_D5_Ct_: 10.74 ± 1.02, *n* = 88; *p* < 0.001) and the explored area was larger (Figure 4F, control: 0.30 ± IQR 0.02 to 1.17 10^−2^ µm^2^/s, *n* = 448; _TAT_D5_Ct_: 1.33 ± IQR 0.60 to 3.96, *n* = 77; *p* < 0.001).

**Figure 5:**
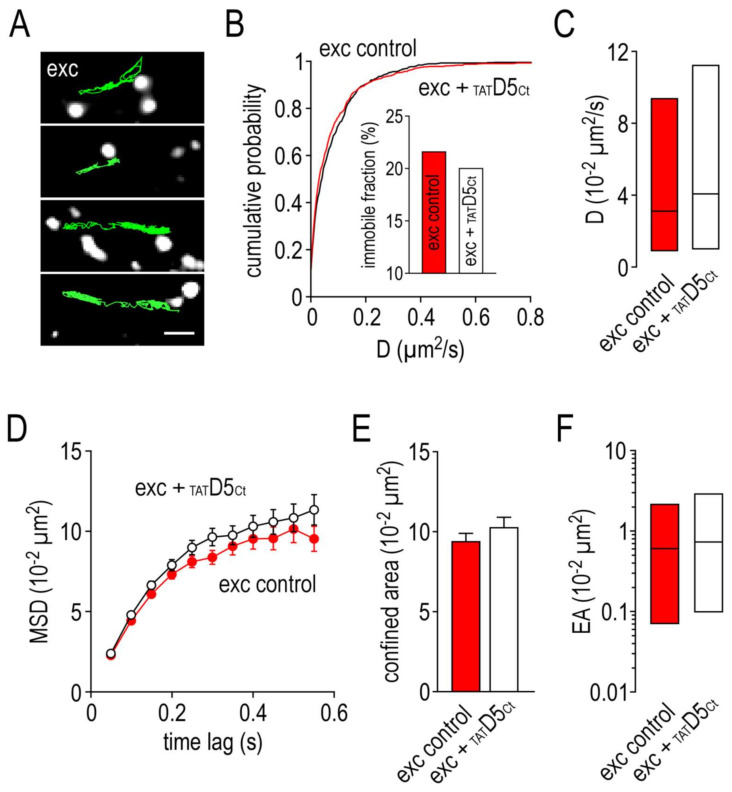
The disrupting peptide has no impact on the surface dynamic properties of GABA_A_ receptors at excitatory synapses. (**A**) Examples of trajectories of a single GABA_A_ receptor–SEP–anti-GFP antibody–QD complex (500 frames, 50 ms acquisition) in the presence of the competing peptide (10 µM) with identified excitatory (exc) synaptic areas. Scale bar, 350 nm. (**B**) Cumulative probability diffusion coefficient of GABA_A_ receptors at excitatory synapses in control condition or in the presence of the disrupting peptide. The inset shows the immobile fraction (defined as D < 0.005 µm^2^/s) in the excitatory area in control condition and in the presence of the disrupting peptide. (**C**) Bar graphs illustrating the instantaneous diffusion coefficient (D, represented as median ± interquartile range 25–75%) in excitatory area in control condition or in the presence of the disrupting peptide. (**D**) Comparison of GABA_A_ receptors mean square displacements (MSD, represented as mean ± SEM) in excitatory area in control condition or in the presence of the disrupting peptide. (**E**) Bar graphs illustrating the confined area (for the time period of 250–550 ms and represented as mean ± SEM) in excitatory area in control condition or in the presence of the disrupting peptide. (**F**) Bar graphs illustrating the explored surface area (EA, represented as median ± interquartile range 25–75%) in excitatory area in control condition or in the presence of the disrupting peptide.

By contrast, Figure 5A shows that incubating hippocampal neurons with the disrupting peptide had no major impact on the surface dynamic properties of SEP-γ2-GABA_A_Rs located at the excitatory synapses. We observed no changes in the distribution of the diffusion coefficient (Figure 5B), the immobile fraction (Figure 5B inset, control: 22% vs. _TAT_D5_Ct_: 20%), as well as the diffusion coefficient median (Figure 5C, control: 3.15 ± IQR 0.91 to 9.41 10^−2^ µm^2^/s, *n* = 830; _TAT_D5_Ct_: 4.12 ± IQR 1.05 to 11.33, *n* = 598, *p* = 0.13). The confinement level was not impacted by the disrupting peptide either (Figure 5D). Indeed, both the confined area (Figure 5E, control: 9.48 ± 0.46 10^−2^ µm^2^, *n* = 611; _TAT_D5_Ct_: 10.38 ± 0.53, *n* = 458, *p* = 0.095) and the explored area (Figure 5F, control: 0.60 ± IQR 0.07 to 2.10 10^−2^ µm^2^, *n* = 535; _TAT_D5_Ct_: 0.73 ± IQR 0.10 to 2.83, *n* = 388; *p* = 0.097) remained unchanged.

**Figure 6:**
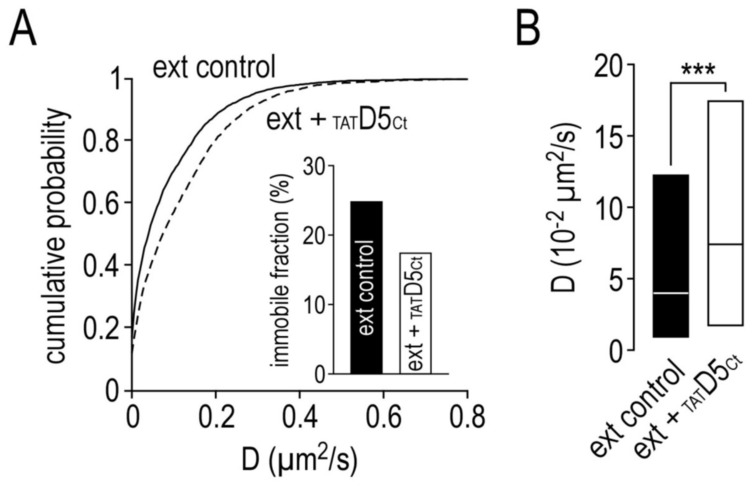

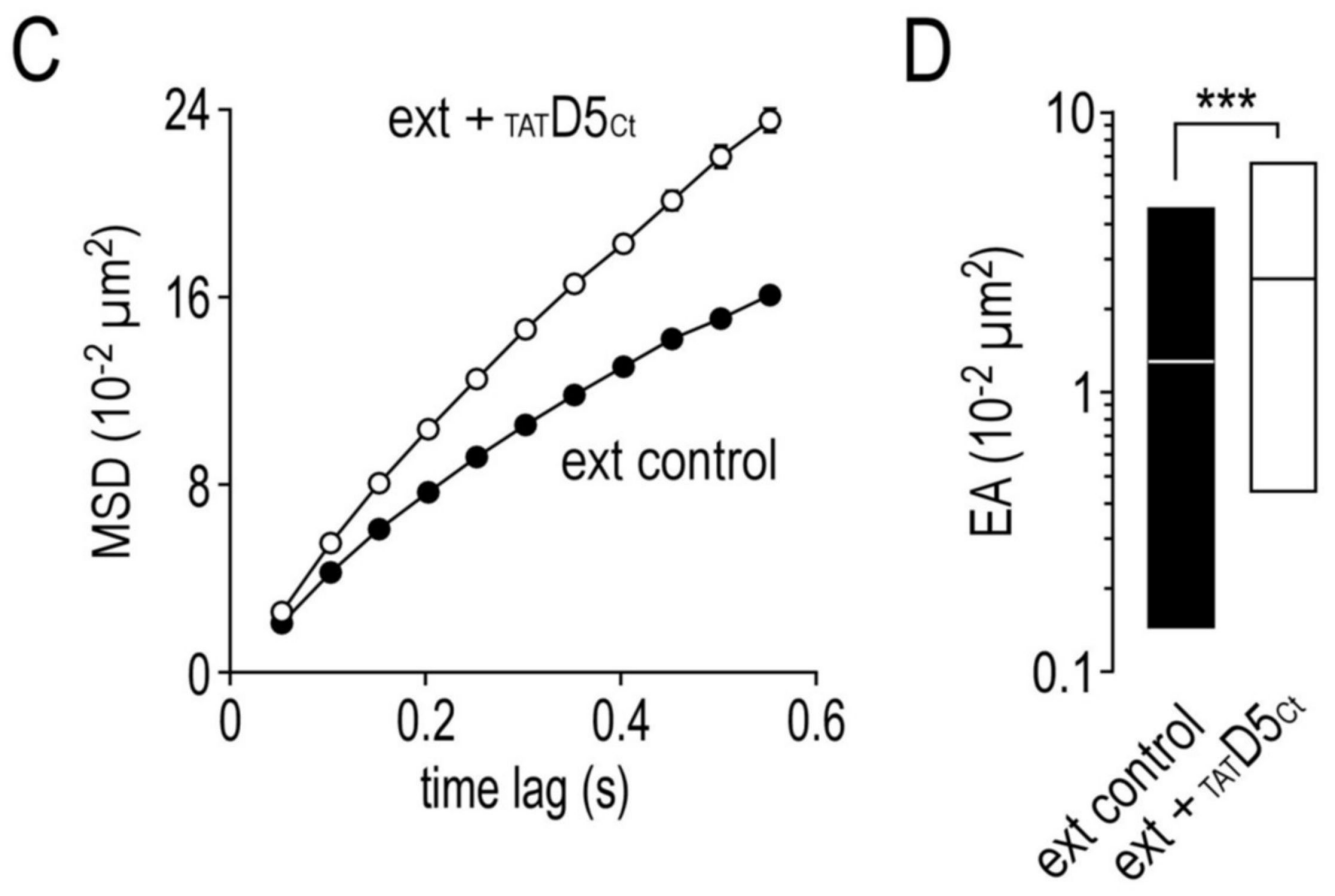
The disrupting peptide also affects the surface dynamic properties of extrasynaptic GABA_A_ receptors. (**A**) Cumulative probability diffusion coefficient of extrasynaptic GABA_A_ receptors in control condition or in the presence of the disrupting peptide (10 µM). The inset shows the immobile fraction (defined as D < 0.005 µm^2^/s) of extrasynaptic GABA_A_ receptors in control condition and in the presence of the disrupting peptide. (**B**) Bar graphs illustrating the instantaneous diffusion coefficient (D, represented as median ± interquartile range 25–75%) of extrasynaptic GABA_A_ receptors in control condition or in the presence of the disrupting peptide. (**C**) Comparison of extrasynaptic GABA_A_ receptors mean square displacements (MSD, represented as mean ± SEM) in control condition or in the presence of the disrupting peptide. (**D**) Bar graphs illustrating the explored surface area (EA, represented as median ± interquartile range 25–75%) of extrasynaptic GABA_A_ receptors in control condition or in the presence of the disrupting peptide. *** *p* < 0.001. Nonparametric Mann–Whitney test.

Lastly, the extrasynaptic SEP-γ2-GABA_A_R surface dynamics were also modulated by the disrupting peptide (Figure 6). Figure 6A shows that the extrasynaptic SEP-γ2-GABA_A_R diffusion was higher after the disrupting peptide incubation, as indicated by the left shift of the distribution curves. Consequently, the immobile fraction of the extrasynaptic receptors was smaller (Figure 6A inset, control: 25%; _TAT_D5_Ct_: 17%) and the diffusion coefficient median (Figure 6B, 4.11 ± IQR 0.73 to 12.20 10^−2^ µm^2^/s, *n* = 4339) was significantly increased after the disrupting peptide incubation (7.50 ± IQR 1.78 to 17.50 10^−2^ µm^2^/s, *n* = 3072, *p* < 0.001). Finally, Figure 6C shows that the extrasynaptic SEP-γ2-GABA_A_Rs that are already poorly confined became even less confined (control: 12.59 ± 0.22 10^−2^ µm^2^, *n* = 4196; _TAT_D5_Ct_: 17.64 ± 0.31, *n* = 2974, *p* < 0.001) and they explored an even larger area (Figure 6D, control: 1.24 ± IQR 0.14 to 4.40 10^−2^ µm^2^, *n* = 4110; _TAT_D5_Ct_: 2.45 ± IQR 0.45 to 6.60, *n* = 2926; *p* < 0.001). Altogether, these data indicate that the interaction between the γ2-GABA_A_R and D5R strongly structures the dynamics of the GABA_A_Rs at inhibitory—not at glutamatergic—synapses.

### 2.6. Surface Dynamics Properties of Dopamine D5 Receptors

**Figure 7:**
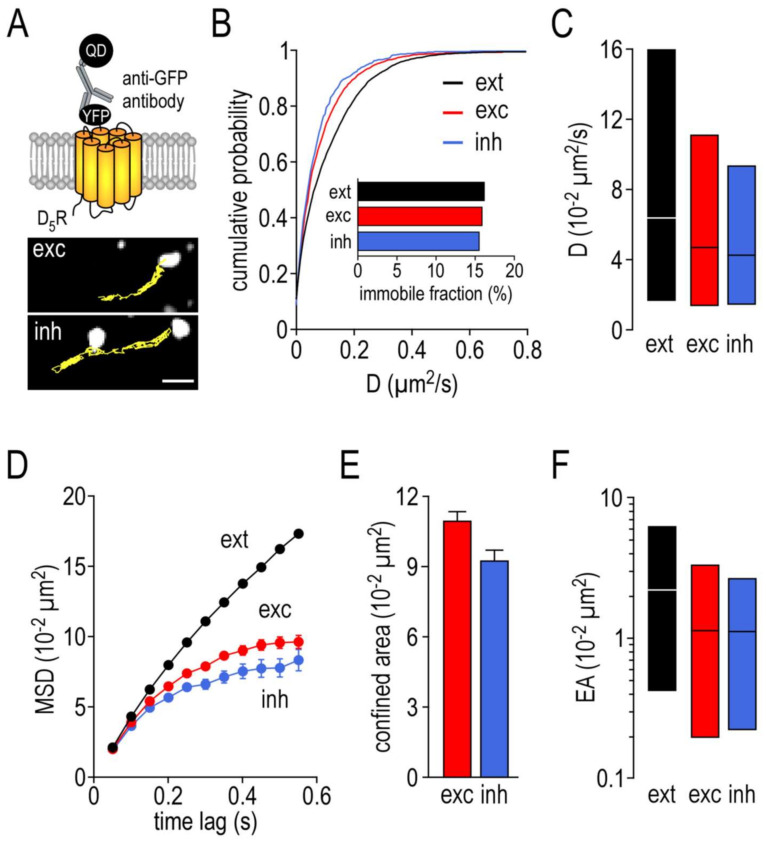
The surface dynamic properties of dopamine D5 receptors are not differentially altered at excitatory or inhibitory synapses. (**A**) Cartoon showing the experimental design (upper panel) and examples of trajectories of a single dopamine D5 receptor–YFP–anti-GFP antibody–QD complex (500 frames, 50 ms acquisition) on the dendritic shaft with either identified excitatory (exc) or inhibitory (inh) synaptic areas (lower panel). Scale bar, 350 nm. (**B**) Cumulative probability diffusion coefficient of dopamine D5 receptors at inh or exc synapses compared to extrasynaptic area (ext). The inset shows the immobile fraction (defined as D < 0.005 µm^2^/s) in the different areas. (**C**) Bar graphs illustrating the instantaneous diffusion coefficient (D, represented as median ± interquartile range 25–75%) of dopamine D5 receptors in ext, exc and inh areas. (**D**) Comparison of dopamine D5 receptors mean square displacements (MSD, represented as mean ± SEM) in ext, exc and inh areas. (**E**) Bar graphs illustrating the confined area (for the time period 250–550 ms and represented as mean ± SEM) of dopamine D5 receptors in exc and inh areas. (**F**) Bar graphs illustrating the explored surface area (EA, represented as median ± interquartile range 25–75%) of dopamine D5 receptors in ext, exc and inh areas.

As illustrated in the Figure 7A upper panel, the N-terminal domain of the receptor was fused to the yellow fluorescent protein (YFP). The complex formed by the membrane receptor–anti-GFP antibody–quantum dots was then tracked, and the trajectories correlated to either the excitatory or inhibitory synapses by co-transfecting the neurons with homer1c–DsRed and gephyrin–mRFP, respectively (Figure 7A, bottom panel). As previously described with SEP-γ2-GABA_A_Rs, the diffusion of D5Rs was lower at the synaptic sites as indicated by the left shift of the distribution curves (Figure 7B). Consistently, the extrasynaptic D5R diffusion coefficient median was high (6.42 ± IQR 1.73 to 16.00 10^−2^ µm^2^/s, *n* = 6182) and decreased at synaptic sites (Figure 7C). Contrary to SEP γ2-GABA_A_Rs, no distinction could be made between the excitatory (4.72 ± IQR 1.41 to 11.10 10^−2^ µm^2^/s, *n* = 1602) and inhibitory (4.29 ± IQR 1.47 to 9.30 10^−2^ µm^2^/s, *n* = 545, *p* = 0.15) synapses. Interestingly, the immobile fraction was low within both synapses (exc: 16.1%; inh: 15.6%) and close to the immobile fraction of the extrasynaptic receptors (16.3%), suggesting that D5Rs were not anchored or stabilized at synapses (Figure 7B inset). Yet, the synaptic D5Rs were more confined compared to the extrasynaptic receptors (Figure 7D). However, as illustrated in Figure 7E, this level of confinement is rather low and indistinguishable between the excitatory (11.06 ± 0.33 10^−2^ µm^2^, *n* = 1208) and inhibitory (9.31 ± 0.45 10^−2^ µm^2^, *n* = 410, *p* = 0.12) synapses. Figure 7F confirms that the explored area of the extrasynaptic D5Rs is large (median: 2.13 ± IQR 0.42 to 5.97 10^−2^ µm^2^, *n* = 5786) and only slightly reduced at the synaptic areas. Together, this membrane dynamic shows no preference between excitatory (median: 1.10 ± IQR 0.20 to 3.24 10^−2^ µm^2^, *n* = 1041) and inhibitory (median: 1.09 ± IQR 0.22 to 2.54 10^−2^ µm^2^, *n* = 344) synapses (*p* = 0.23).

### 2.7. Impact of the Disrupting Peptide on the Surface Dynamics of Dopamine D5 Receptors

**Figure 8:**
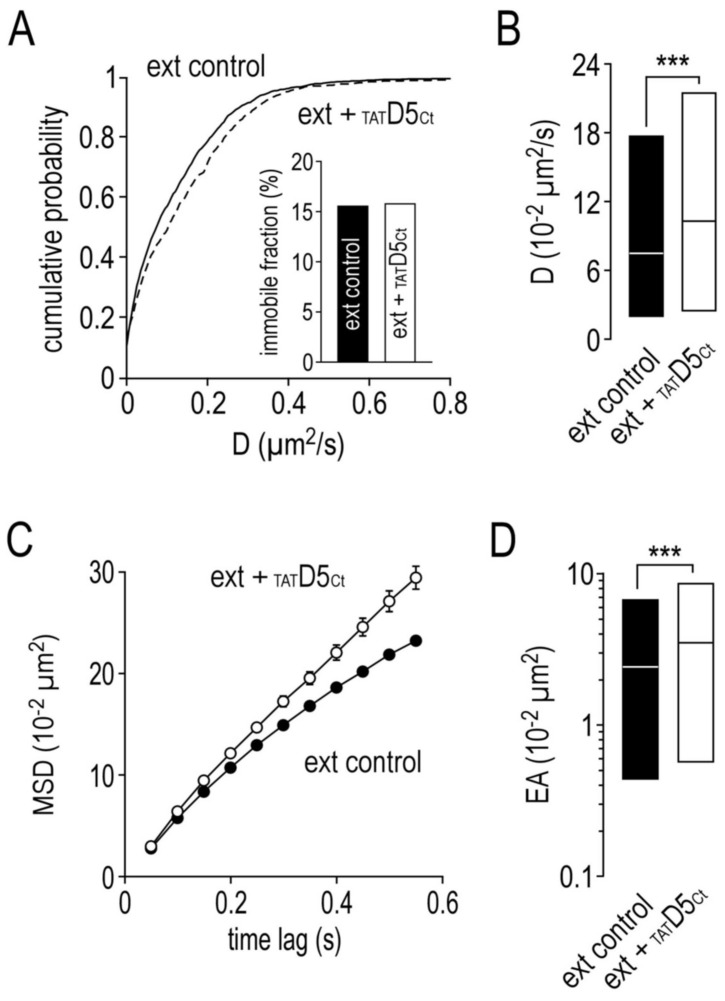
The disrupting peptide impairs the surface dynamic properties of extrasynaptic dopamine D5 receptors. (**A**) Cumulative probability diffusion coefficient of extrasynaptic dopamine D5 receptors in control condition or in the presence of the disrupting peptide (10 µM). The inset shows the immobile fraction (defined as D < 0.005 µm^2^/s) of extrasynaptic dopamine D5 receptors in control condition and in the presence of the disrupting peptide. (**B**) Bar graphs illustrating the instantaneous diffusion coefficient (D, represented as median ± interquartile range 25–75%) of extrasynaptic dopamine D5 receptors in control condition or in the presence of the disrupting peptide. (**C**) Comparison of extrasynaptic dopamine D5 receptors mean square displacements (MSD, represented as mean ± SEM) in control condition or in the presence of the disrupting peptide. (**D**) Bar graphs illustrating the explored surface area (EA, represented as median ± interquartile range 25–75%) of extrasynaptic dopamine D5 receptors in control condition or in the presence of the disrupting peptide. *** *p* < 0.001. Nonparametric Mann–Whitney test.

As dopamine D5 receptors are mostly located outside the synapses and don’t seem to be retained at particular synapses, we focused our investigation on the effects of the disrupting peptide on extrasynaptic D5Rs. D5R diffusion was higher after the disrupting peptide incubation, as indicated by the left shift of the distribution curves (Figure 8A). Noteworthy, this shift does not change the immobile fraction (Figure 8A inset), which remains stable at around 15%. The D5R diffusion coefficient median (Figure 8B, 7.42 ± IQR 1.91 to 17.70 10^−2^ µm^2^/s, *n* = 2086) was significantly increased after the disrupting peptide incubation (10.30 ± IQR 2.42 to 21.37 10^−2^ µm^2^/s, *n* = 928, *p* < 0.001). With the disrupting peptide, the D5Rs became even less confined (control: 21.9 ± 0.3 10^−2^ µm^2^, *n* = 4224; _TAT_D5_Ct_: 26.9 ± 0.9, *n* = 721, *p* < 0.001) and explored an even larger area (control: 2.37 ± IQR 0.44 to 8.50 10^−2^ µm^2^, *n* = 5152; _TAT_D5_Ct_: 3.45 ± IQR 0.57 to 8.50, *n* = 873; *p* < 0.001) (Figure 8C,D). Thus, these data indicate that the D5R dynamics are slightly regulated by its interaction with the γ2-GABA_A_R.

### 2.8. Functional Consequences of the Disruption of GABA_A_R–D5R Complexes

**Figure 9:**
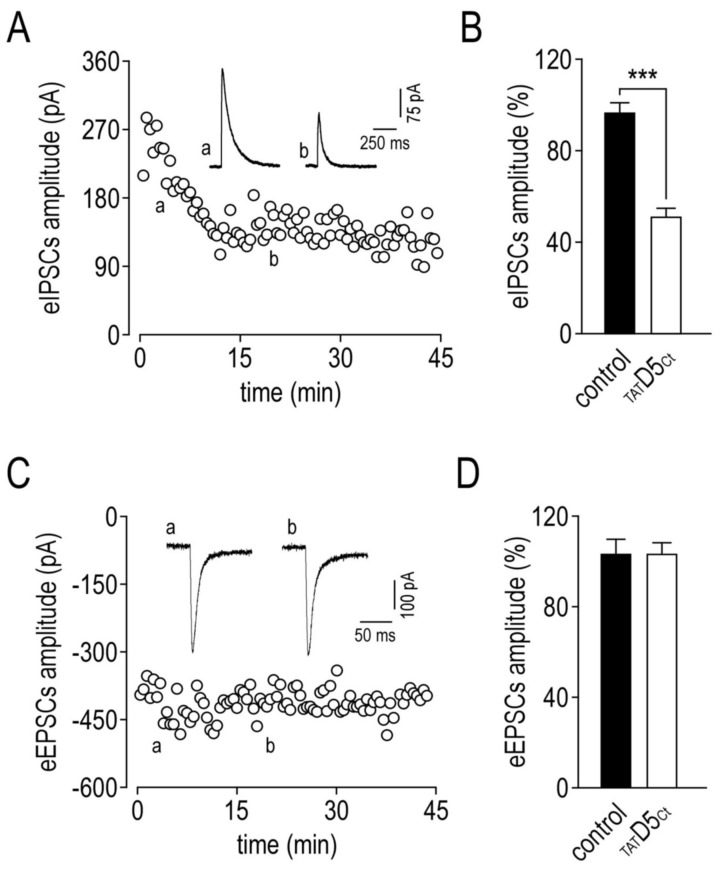
Evoked GABA_A_ receptor-mediated IPSCs recorded in hippocampal CA1 pyramidal neurons are depressed by the disrupting peptide. (**A**) eIPSCs amplitude versus time (recorded at a holding potential of −40 mV) with the disrupting peptide added in the patch pipette (10 µM). The inset shows example traces of eIPSCs two minutes after the achievement of the whole-cell configuration (a), and at the maximal time effect of the disrupting peptide (b). For this figure and the next, stimulus artefacts were digitally removed. (**B**) Summary data comparing the effects of control and disrupting peptide on eIPSCs amplitude (represented as mean ± SEM at t = 20 min, normalized to the amplitude at t = 2 min. *** *p* < 0.001. Nonparametric Mann–Whitney test). Experiments in the presence of 50 µM D-AP5 and 2 µM NBQX. (**C**) Evoked AMPA receptor-mediated EPSCs amplitude versus time (recorded at a holding potential of −70 mV) with the disrupting peptide added into the patch pipette (10 µM). The inset shows example traces of eEPSCs 2 min (a) and 20 min (b) after the achievement of the whole-cell configuration. (**D**) Summary data comparing the effects of control and disrupting peptide on eEPSCs amplitude (represented as mean ± SEM at t = 20 min, normalized to the amplitude at t = 2 min). Experiments in the presence of 10 µM bicuculline.

We then tested whether the rapid redistribution of SEP γ2-GABA_A_Rs (caused by the disrupting peptide) outside synapses impacts the GABAergic transmission. For this, we recorded the inhibitory post-synaptic currents (IPSCs) from hippocampal CA1 pyramidal neurons. Monosynaptic IPSCs were evoked by the local electrical stimulation of GABAergic axons in stratum radiatum from neurons voltage-clamped at a holding potential of −40 mV in hippocampal slices (prepared from 12–14-day-old rats). The outwardly going evoked IPSCs were pharmacologically isolated in the presence of 50 µM D-AP5 and 2 µM NBQX (Figure 9A, inset). Once the whole-cell configuration was achieved, the disrupting peptide, added in the patch pipette (10 µM), was able to diffuse into the cell body of the neuron and it produced a rapid depression in the recorded evoked IPSC amplitude (Figure 9A,B, 51.7 ± 3.3%, *n* = 11, ***, *p* = 0.0002), while under control conditions the evoked IPSC amplitude remained stable (Figure 9B, 97.1 ± 4.4%, *n* = 6). Next, we applied the same experimental protocol on the excitatory post-synaptic AMPA receptor-mediated currents (EPSCs) to test whether the increased lateral diffusion of GABA_A_Rs could alter the functioning of the nearby excitatory synapses. Inwardly going evoked EPSCs were therefore recorded at −70 mV and pharmacologically isolated in the presence of 10 µM bicuculline (Figure 9C, inset). In these experiments, neither the control (104.0 ± 5.9%, *n* = 6) nor the disrupting peptide were able to alter the evoked EPSC amplitude (104.1 ± 4.4%, *n* = 6, *p* = 0.94), suggesting that the lateral redistribution of GABA_A_Rs is not able to affect the functional synaptic AMPA receptors (Figure 9C,D).

**Figure 10:**
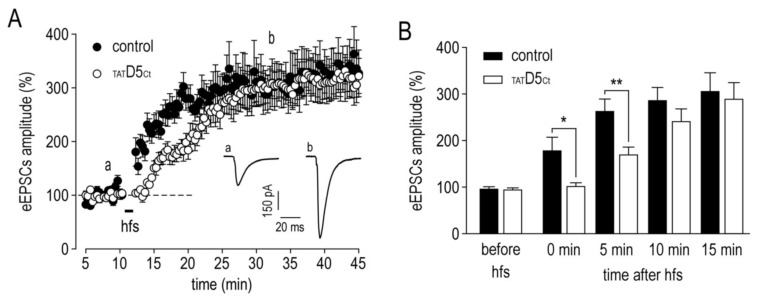
The disrupting peptide shifts in time the expression of long-term potentiation at CA3–CA1 synapse in hippocampal slices. (**A**) Pooled data showing eEPSCs amplitude versus time for all experiments in which control or disrupting peptide was added in the patch pipette. The inset shows representative traces of eEPSCs before (a) the high-frequency stimulation (hfs) and after (b) the expression of the LTP. (**B**) Summary bar graph showing pooled data for the effects of control or disrupting peptide at various time points after hfs. Data are represented as mean ± SEM normalized to the mean amplitude of eEPSCs before hfs. * *p* < 0.05, ** *p* < 0.01. Nonparametric Mann–Whitney test. Experiments in the presence of 10 µM bicuculline.

Finally, we decided to test whether this lateral redistribution of the GABA_A_Rs and the reduced GABAergic synaptic currents would impair the establishment of NMDA receptor-dependent long-term potentiation (LTP). In the control condition, a high-frequency stimulation (hfs) of Schaffer collaterals induced a rapid and persistent increase in the evoked EPSC amplitude (Figure 10A). Right after the hfs (first minutes), a potentiation immediately took place (Figure 10B, EPSCs amplitude: 180.7 ± 26.5%, *n* = 9), whereas this was not observed in the presence of the disrupting peptide (EPSCs amplitude: 104.1 ± 5.1%, *n* = 11, *p* = 0.012). This difference still occurred 5 min after the hfs (control: 265.5 ± 23.8%, *n* = 9; _TAT_D5_Ct_: 171.8 ± 14.3%, *n* = 11, *p* = 0.003). Ten minutes after the hfs, the effect of the disrupting peptide was no longer observed and the evoked EPSC amplitude was similar in both conditions (control: 288.2 ± 25.5%, *n* = 9; _TAT_D5_Ct_: 243.6 ± 24.1%, *n* = 11, *p* = 0.13). These data indicate that the interaction between the γ2-GABA_A_R and the D5R reduces GABAergic transmission and significantly alters the timing of NMDA receptor-dependent LTP at the excitatory synapses.

## 3. Discussion

Using a combination of imaging and single nanoparticle tracking, here we provide evidence that the GABA_A_R assembles with the D5R at the surface of hippocampal neurons, as originally described by biochemical means [28]. We have carried out an extensive characterization of GABA_A_Rs and D5Rs surface dynamics and have demonstrated that both receptors are more mobile and explore a larger area following the disruption of the complex by a competing peptide. The latter also leads to a drop in immobile synaptic GABA_A_Rs, correlated by means of patch-clamp recording, and a robust decrease in GABAergic synaptic current. As a further consequence, we also report a delay in the expression of LTP at the glutamatergic synapses.

Single nanoparticle imaging demonstrates that both GABA_A_Rs and D5Rs are highly dynamic at the surface of hippocampal neurons. GABA_A_Rs show a confined behavior, a substantially slower diffusion and a higher fraction of immobilization at matched (inhibitory) and mismatched (excitatory) synapses. This counterintuitive feature has already been described and well explained by the presence of obstacles and fences (i.e., molecular crowding, lipid composition or cytoskeleton elements) in synapses, which significantly hindered the lateral diffusion of receptors [35]. Our data further confirm that GABA_A_R diffusion slowdown is more pronounced at the inhibitory synapses which contain the scaffolding apparatus favoring their anchoring. Although D5Rs are highly diffusive when entering synapses, they share the typical reduced dynamics no matter the synaptic nature (excitatory versus inhibitory). We do not provide evidence of a significant slowdown of D5Rs at inhibitory synapses, strongly advocating the absence of specific anchoring proteins for D5Rs at these synapses. Interestingly, a similar conclusion was drawn from the single nanoparticle tracking of NMDAR–D1R complexes which suggests that it is not specific to GABA_A_R–D5R interaction [34]. Indeed, a physical interaction has been described between D1R, the other member of the D1-like family, and NMDAR, a major actor in glutamatergic synaptic transmission [36]. This interaction is governed by a dual protein–protein interaction between the C-terminal domain of the D1R and the C-terminal domain of the GluN1 and/or the GluN2A subunits. Functionally, these NMDAR–D1R interactions are involved in the inhibition of NMDAR-mediated currents and the attenuation of NMDAR-mediated excitotoxicity [36]. In addition, the NMDAR–D1R physical interaction modulates NMDAR-dependent synaptic transmission through the lateral redistribution of NMDARs [34]. But, even though D1Rs assemble with NMDARs, they are more mobile than NMDARs and they are not anchored at the excitatory synapses. Instead, the D1Rs and NMDARs form dynamic surface clusters in the vicinity of the excitatory synapses [34]. In that line, similar to what we described with the D5Rs, no distinction is made in the D1R surface dynamics between the excitatory and inhibitory synapses (unpublished data).

With a peptide mimicking the last amino acids of the C-terminal domain of the D5R, we are able to disrupt this interaction. In the original study, this amino acid sequence was demonstrated to bind the second intracellular loop of the γ2-GABA_A_R subunit [28]. Noteworthy, this γ2-GABA_A_R subunit is of particular interest as it also physically interacts with the GABA_B_ receptor [37], the only other example of a physical interaction between the GABA_A_R and other receptors. This disrupting peptide strategy was already successful to demonstrate the functional cross-talk between the D1R and NMDAR [34,36]. In our study, disrupting the GABA_A_R–D5R interaction leads to a significant increase in the diffusion of GABA_A_Rs, which laterally redistribute to larger areas. This has no impact on the properties of GABA_A_Rs at mismatched synapses, as diffusing GABA_A_Rs under normal circumstances only pass the post-synaptic densities where they do not accumulate. However, the receptors higher diffusion impacts the anchoring process at the inhibitory synapses as the immobile fraction of the GABA_A_Rs drops drastically. The D5Rs were also more mobile and explored a larger area following the GABA_A_R–D5R complex disruption, with no further consequence on the immobile fraction which was already low in the control condition anyway. As the competing peptide leads to an increase in the GABA_A_Rs surface dynamics and a decrease in the immobile synaptic receptors, one can expect functional consequences in synaptic transmission. Indeed, disrupting GABA_A_R–D5R interaction leads to a decrease in the GABA_A_R-mediated postsynaptic currents. Although the dissociation rate of this protein–protein interaction is unknown, we suggest that it is rather low based on the time scale of both single nanoparticle tracking and patch-clamp experiments. The large lateral redistribution of GABA_A_Rs and the reduction in the anchoring mechanisms could explain the decrease in the GABA_A_R-mediated postsynaptic currents. Altogether, this demonstrates that, at the level of the plasma membrane, dopamine can differentially modulate two distinct and opposite neurotransmitters synaptic functions through its binding to D1-like receptors, independently from the GPCRs’ classical transduction pathways. Any impairments of this dialogue between dopamine, the NMDAR and the GABAAR, which rely on intracellular protein kinase signaling cascades, has been linked to neurodevelopmental psychiatric disorders such as schizophrenia [38]. When first characterized, the GABAAR–D5R interaction was found to be agonist-dependent [28]. However, to date we are still lacking specific pharmacological tools to discriminate the D1R and D5R, and studies on this dopamine receptor subfamily are greatly hindered. As a consequence, despite its widespread expression in the brain [31,32], D5R intracellular signaling remains poorly characterized, and in most cases regulatory mechanisms are attributed to D1-like receptors. Our results shed new light on the functional role of the GABAAR–D5R interaction, and given that both receptors may be implicated in psychiatric disorders, the GABAAR–D5R complex might be of interest as a new therapeutic target. This approach, which targets the receptor–receptor interaction instead of the receptors alone, has already been successful with the dopamine D2R and DISC1 (disrupted in schizophrenia1), a scaffolding protein involved in psychiatric diseases [39]. The authors demonstrated that disrupting the D2R–DISC1 complex improved the efficacy and reduced the sides effects compared to classical drugs which target receptors alone. The same disrupting strategy targeting the D2R and the dopamine transporter (DAT) restored locomotor activity in control and dopamine-depleted rats by increasing the extracellular dopamine, and exerted beneficial effects in a rat model of attention-deficit hyperactivity disorder [40].

Our data also show that disrupting the GABA_A_R–D5R complexes alter the expression of LTP at glutamatergic synapses during the first minutes following the induction protocol. To our knowledge, this is a peculiar effect as most of the known alterations of LTP either occluded or impacted its amplitude [41,42]. An attractive hypothesis would be that, once the interaction between the GABA_A_R and D5R is prevented, they become highly mobile, exit inhibitory synapses and more likely enter the neighboring excitatory synapses. This would upregulate the molecular crowding in these synapses and eventually impair the lateral recruitment of AMPARs following LTP induction [43,44,45]. Over time and since there is no scaffolding proteins to anchor the GABA_A_Rs at the glutamatergic synapses, the AMPARs will stably anchor and increase their numbers. Several lines of evidence may support this hypothesis. It is now well accepted that the lateral diffusion of surface receptors and their transient anchoring at synapses is regulated by neuronal activity and plays a key role in synaptic plasticity [46]. For example, at excitatory synapses, the recovery from the fast synaptic depression of AMPA receptor-mediated currents is mediated through lateral diffusion and the fast exchange of synaptic desensitized receptors with naïve extrasynaptic ones [47]. Similarly, extrasynaptic α5-GABA_A_Rs provide a reservoir for the rapid supply of receptors into the GABAergic synapses involved in tonic inhibition and GABAergic synaptic transmission [48]. The idea that the lateral diffusion of surface receptors could interfere with neighboring synapses has rapidly emerged. Indeed, it has been shown that after high stimulation of GABAergic synapses, desensitized GABA_A_Rs laterally diffuse to nearby GABAergic synapses and reduce the GABA_A_R-mediated currents [49]. This phenomenon, based on the disponibility of desensitized receptors and the time required for them to reach the neighboring synapse, opens new research avenues on GABAergic synaptic transmission and plasticity. Indeed, GABA_A_Rs can enter long-lasting desensitized states following high-frequency stimulation, which favor diffusion over few micrometers contrary to AMPA receptors which recover quickly from inactivation [50,51]. It is therefore unlikely that desensitized AMPA receptors exit postsynaptic spines and reach other glutamatergic synapses, so this peculiar mechanism of intersynaptic cross-talk might be specific to the inhibitory synapses. A step further has recently been taken with the evidence that LTP induction not only induces the insertion of AMPARs but also allows α5-GABA_A_Rs to laterally diffuse and get trapped at GABAergic synapses, preventing consequently additional LTP [52]. Altogether, this new evidence uncovers a non-canonical role of receptors surface dynamics in the regulation of synaptic physiology. Further investigations are now needed to demonstrate, more specifically, the role of the GABA_A_R–D5R interaction on short-term and long-term GABAergic synaptic plasticity.

Finally, GABA, as a neurotransmitter, plays a crucial role in synaptic maturation. In the developing hippocampus, the GABAergic synapses are the first functional ones, followed by the glutamatergic synapses [53]. GABA is even more important because of the developmental changes in chloride homeostasis due to the differential expression of chloride cotransporters [54]. These characteristics explain the developmental switch in the action of GABA, from depolarizing to hyperpolarizing. Since GABA_A_Rs have a pivotal role during synaptic development and the functional cross-talk with dopamine receptors, one can wonder whether dopamine would modulate the inhibitory synaptogenesis. In developing striatum, dopamine decreases the number of functional GABAergic synapses formed between the embryonic precursors of the medium spiny neurons and therefore changes their spontaneous synaptic activity [55]. However, the functional role of the GABA_A_R–D5R interaction in inhibitory synaptogenesis remains an uncharted field. It is likely that dopamine exerts its key developmental actions through GABA_A_R internalization via dephosphorylation processes. It is also worth noting that regional differences in the GABA_A_R–D5R interaction may take place, as one study was unable to demonstrate the physical interaction in the striatum [56]. In conclusion, our study provides evidence of a functional cross-talk between the GABA_A_R and D5R in hippocampal neurons. This sheds new light on the dopamine regulatory mechanisms on GABAergic synaptic physiology and sets the surface protein–protein interactions at the primary stage of signal integration. Further investigations are now clearly needed to fully uncover whether this functional cross-talk is specific to hippocampal neurons, and whether it occurs early in development and has a functional role in synaptic maturation.

## 4. Material and Methods

All experiments were carried out in accordance with the University of Bordeaux guidelines and regulations. The animal procedures were approved by the ethical committee of the University of Bordeaux.

### 4.1. Hippocampal Neuron Culture

The classical protocol previously described was slightly adapted to prepare primary cultures of hippocampal neurons [57]. Briefly, hippocampi were dissected from embryonic day 18 or 19 Sprague–Dawley rats (Janvier Labs, France), incubated in trypsin-EDTA (0.05%, 15 min at 37 °C) and mechanically dissociated in Hank’s balanced salt solution supplemented with Hepes (HBSS, 10 mM). Neurons (6.25 × 10^4^) were cultured on poly-L-lysine pre-coated (1 mg/mL) 18 mm diameter glass coverslips in Neurobasal medium supplemented with NeuroCult SM1 (2%) and L-glutamine (2 mM). Neurons are kept at 37 °C in 5% CO_2_ until the day of experiments. All products were obtained from ThermoFisher Scientific (Waltham, MA, USA) except SM1 (Stem Cell Technologies, Vancouver, Canada) and poly-L-lysine (Sigma-Aldrich, St. Louis, MO, USA).

### 4.2. Peptides Synthesis and Their Experimental Use

Synthetized peptides were purchased from Caslo (Lyngby, Denmark) and rendered cell-permeable by fusing the transduction domain sequence from the human immunodeficiency virus TAT protein (GRKKRRQRRR). The disrupting peptide (_TAT_D5_Ct_) contains the sequence of the last amino acids of the C-terminal domain of the dopamine D5 receptor (F429 to A475). The control peptide consists in a scramble sequence of the same amino acids. All peptides were used at a final concentration of 10 µM. They were incubated with the neurons for 45 min prior to fixation in immunocytochemistry experiments. They were incubated with the neurons for 10 min before the incubation of the antibodies and the quantum dots. In patch-clamp experiments, peptides were added to the intracellular medium in the patch pipette.

### 4.3. Immunocytochemistry Experimental Protocols

Prior to being cultured on coverslips, neurons were electroporated with plasmids coding for the recombinant γ2-GABA_A_ subunits fused either to a superecliptic pHluorin (SEP) or a myc tag, and the D5R fused to the yellow fluorescent protein (YFP) as requested (Nucleofactor technology, Lonza, Basel, Switzerland). At 14–16 days in vitro (DIV), surface receptors were specifically stained (10 min at 37 °C in Neurobasal medium) using antibodies against the respective tags (rabbit anti-GFP 1:500, ThermoFisher Scientific; rabbit anti-myc 1:100, Abcam, Cambridge, UK). Neurons were fixed with paraformaldehyde (4%, 15 min), quenched in ammonium chloride (50 mM, 10 min), permeabilized with Triton X-100 (0.1%, 5 min) and blocked for one hour with bovine serum albumin (BSA, 3%, Sigma-Aldrich). All these steps were carried out in phosphate buffer saline (PBS). A three times wash of 5 min in PBS was performed between all the steps. Subsequently, synapses were specifically stained using mouse anti-gephyrin antibody (1:2000, overnight at 4 °C, Synaptic Systems, Göttingen, Germany) and guinea pig anti-homer1c antibody (1:500, 1 h at room temperature, Synaptic Systems). Dedicated secondary antibodies conjugated to fluorescent dyes (Alexa Fluor^®^ 488, 568 or 637, 1:500, ThermoFisher Scientific) were incubated for one hour at room temperature. These last two steps were carried out in PBS supplemented with BSA (3%). Neurons were mounted in Mowiol (Merck, Kenilworth, NJ, USA). Images were collected on a video confocal spinning disk system DMI6000B (Leica microsystems, Wetzlar, Germany) coupled to a CoolSNAP HQ2 camera (Photometrics, Tucson, AZ, USA) with Metamorph (Molecular devices, San Jose, CA, USA). Clusters analysis was performed using a manual threshold approach based on integrated fluorescence with ImageJ (NIH). For each neuron, between 2 to 4 regions of interest (ROIs) were selected on the dendritic tree and clusters characteristics (intensity, number and area) were averaged out.

### 4.4. Single Nanoparticle Tracking Experimental Protocols

Neurons were transfected at 7–10 DIV using either the Effectene transfection kit according to the manufacturer’s instructions (Qiagen, Hilden, Germany), or the calcium phosphate protocol. According to the dedicated experiments, neurons were transfected with recombinant the γ2-GABA_A_–SEP subunit, the D5R–YFP, homer1c–mDsRed or gephyrin–mRFP (monomeric Red Fluorescent Protein). At 14–16 DIV, neurons were first incubated for 10 min with rabbit anti-GFP antibodies (1:10,000, ThermoFisher Scientific), washed and then incubated for 10 min with F(ab’)2-goat anti-rabbit IgG (H+L) secondary antibodies coupled to quantum dots 655 (QD, 1:100,000, ThermoFisher Scientific). All these steps were performed at 37 °C in Tyrode solution (in mM 108 NaCl, 5 KCl, 25 Hepes, 2 CaCl_2_, 2 MgCl_2_, 15 glucose, pH 7.4 with NaOH) supplemented with BSA (1%). Images and QD recording (500 consecutive frames with an acquisition time of 50 ms) were collected in Tyrode solution (37 °C), on an eclipse T*i* epifluorescence microscope (Nikon) coupled to an Evolve 512 camera (Photometrics) with Metamorph. Single QD tracking and reconstruction of two dimensional trajectories were performed with Metamorph and homemade plugins in MATLAB (Mathworks, Natick, MA, USA) by correlation analysis between consecutive images using Vogel algorithm. This technique provides a high accuracy of single QD detection (~30 nm resolution). The instantaneous diffusion coefficient (D) was calculated for each trajectory from linear fits of the first four points of the mean square displacement (MSD) versus time (t) function using MSD(t) = < r2>(t) = 4 Dt. Receptors were defined as immobile if D < 0.005 µm^2^/s. The explored area (EA) of each trajectory was defined as the MSD value of the trajectory for time intervals between 200 and 300 ms. Trajectories were classified as synaptic when they overlapped (including the surrounding two pixels) with synaptic markers area identified as homer1c–mDsRed or gephyrin–mRFP positive clusters [34,35]

### 4.5. Patch-Clamp Experimental Protocols

Patch-clamp recordings were performed on 12- to 16-day-old Sprague-Dawley rat pups (day 0 is the day of birth). Briefly, animals were deeply anaesthetized using isoflurane, decapitated and transverse hippocampal slices (350 µm thick) were prepared in cooled artificial cerebrospinal fluid (ACSF, in mM: 126 NaCl, 3.5 KCl, 2 CaCl_2_, 1.3 MgCl_2_, 1.2 NaH_2_PO_4_, 25 NaHCO_3_, 12.1 glucose, saturated with 95% O_2_–5% CO_2_), using a vibratome VT 1000S (Leica biosystems, Wetzlar, Germany). Hippocampal slices were stored in ACSF at room temperature. Whole-cell patch-clamp recordings from visually identified CA1 pyramidal neurons were made using electrodes (3–5 MΩ) filled with the following intracellular solution (mM): 134 CsMeSO_4_, 4 NaCl, 10 Hepes, 0.5 EGTA, 4 Mg-ATP, 0.3 Na-GTP, pH 7.2 with CsOH. The extracellular medium was ACSF at 32 °C. Monosynaptic pharmacologically isolated post-synaptic currents were evoked using local extracellular stimulation of Schaffer collaterals in stratum radiatum at a frequency of 0.05 Hz. Inhibitory post-synaptic currents (IPSCs) were recorded at a holding potential of −40 mV in the presence of d(-)-2-amino-5 phosphonopentanoic acid (D-AP5, 50 µM) to block N-methyl-D-aspartate (NMDA) receptors and 1,2,3,4- tetrahydro-6-nitro 2,3-dioxo-benzo[f]quinoxaline-7-sulfonamide (NBQX, 2 µM) to block α-amino-3-hydroxy-5-méthylisoazol-4-propionate (AMPA) receptors. Excitatory post-synaptic currents (EPSCs) were recorded at a holding potential of −70 mV in the presence of bicuculline (10 µM) in order to block GABA_A_ receptors. Long-term potentiation (LTP) was induced by a high-frequency stimulation of Schaffer collaterals (250 stimulations at 2.5 Hz) while depolarizing the postsynaptic neuron to 0 mV. Data were recorded using an Axon MultiClamp 700B amplifier and analyzed using Clampfit 10.7 (Molecular Devices). The access resistance was monitored throughout the experiment and cells were rejected if it changed by >20%. All drugs and salts were respectively obtained from Tocris (Bristol, UK) and Sigma-Aldrich.

## Supplementary Materials

The following are available online at https://www.mdpi.com/article/10.3390/ijms22094867/s1.
