# Supplementary material for: Characterization of the Functional Cross-Talk between Surface GABAA and Dopamine D5 Receptors
Source: Int J Mol Sci. 2021 May 4;22(9):4867. doi: 10.3390/ijms22094867 (PMC8125140; doi:10.3390/ijms22094867)
Supplement: Supplementary file 1 [file ijms-22-04867-s001.zip › ijms-1190015-supplementary.pdf]

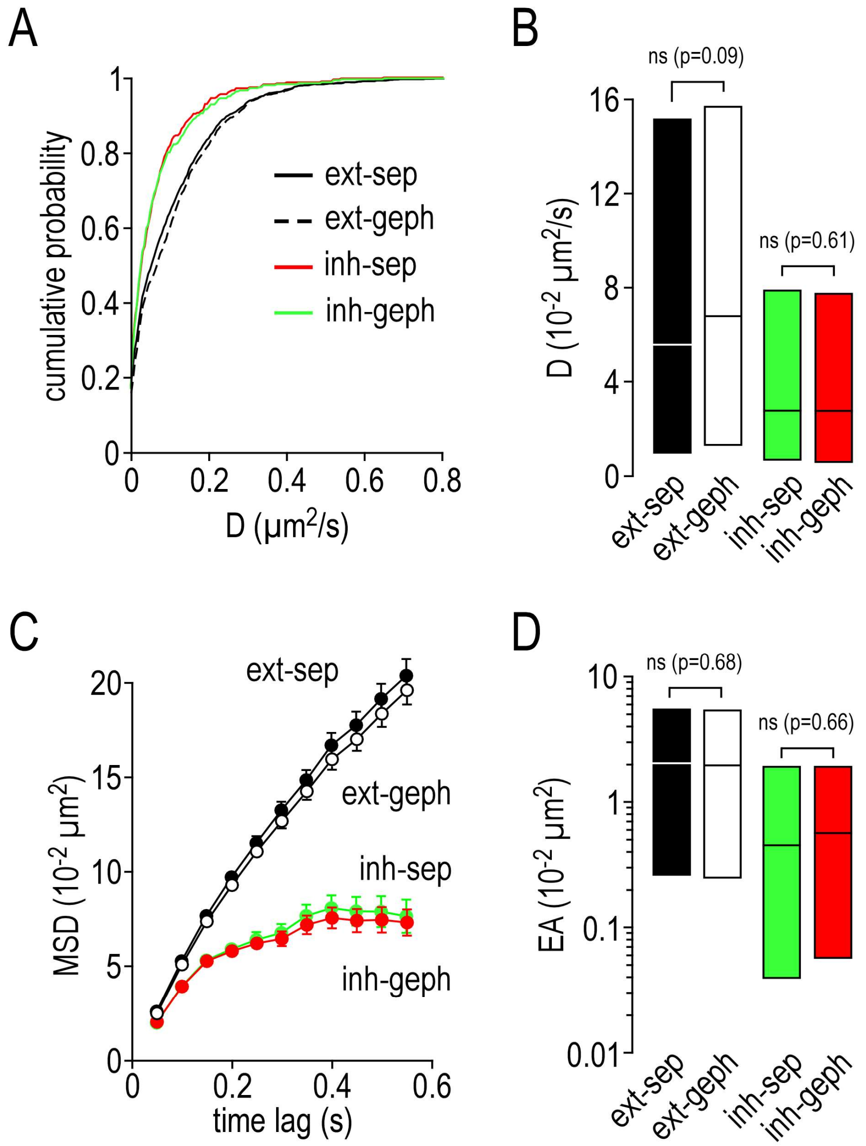

Supplementary figure 1: The surface dynamics properties of GABA$_A$ receptors are identical regardless of how inhibitory postsynaptic sites are identified. These single nanoparticle tracking experiments were performed at DIV14 on cultured hippocampal neurons transfected with γ2-GABA$_A$ subunit fused with a SEP tag and gephyrin fused with a mRFP (18 neurones, 1564 trajectories). **(A)** Cumulative probabilities diffusion coefficient of GABA$_A$ receptors in inhibitory (inh) and extrasynaptic areas (ext) in analyses where inhibitory synapses have been identified using either SEP or mRFP (geph) fluorescence. **(B)** Bar graphs illustrating the instantaneous diffusion coefficient (D, represented as median $\pm$ interquartile range 25-75%) in ext and inh areas for both types of analysis. **(C)** Comparison of GABA$_A$ receptors mean square displacements (MSD, represented as mean $\pm$ SEM) in ext and inh areas for both types of analysis. (D) bar graphs illustrating the explored surface area (EA, represented as median $\pm$ interquartile range 25-75%) n ext and inh areas for both types of analysis. Ns, non-significant, nonparametric Mann-Whitney test.
